# Guidelines for Rational Cancer Therapeutics

**DOI:** 10.3389/fonc.2017.00310

**Published:** 2017-12-12

**Authors:** Byunghee Yoo, Ann-Marie Billig, Zdravka Medarova

**Affiliations:** ^1^MGH/MIT/HMS Athinoula A. Martinos Center for Biomedical Imaging, Massachusetts General Hospital and Harvard Medical School, Boston, MA, United States; ^2^Bouvé College of Health Sciences, Northeastern University, Boston, MA, United States

**Keywords:** imaging, cancer, therapy, rational, nanomedicine

## Abstract

Traditionally, cancer therapy has relied on surgery, radiation therapy, and chemotherapy. In recent years, these interventions have become increasingly replaced or complemented by more targeted approaches that are informed by a deeper understanding of the underlying biology. Still, the implementation of fully rational patient-specific drug design appears to be years away. Here, we present a vision of rational drug design for cancer that is defined by two major components: modularity and image guidance. We suggest that modularity can be achieved by combining a nanocarrier and an oligonucleotide component into the therapeutic. Image guidance can be incorporated into the nanocarrier component by labeling with a specific imaging reporter, such as a radionuclide or contrast agent for magnetic resonance imaging. While limited by the need for additional technological advancement in the areas of cancer biology, nanotechnology, and imaging, this vision for the future of cancer therapy can be used as a guide to future research endeavors.

## Introduction

The recent past has seen an impetus to develop more personalized therapies for cancer, as part of the general concept of precision medicine. In this review, we look at a component of precision medicine focused on the specific design of the therapeutic. We suggest that it is possible to design therapeutics that are fully rational. Namely, we envision rational therapeutics as therapeutics based entirely on predesigned components. This strategy is different than strategies that we have seen in the past or are seeing currently. Traditional approaches are largely reliant on trial-and-error discovery. A prime example is penicillin, which was discovered by pure chance. Even more rational modern approaches such as combinatorial screening, have a large element of trial-and-error in their discovery. The approach we are talking about has no trial-and-error components. It is entirely predesigned, meaning that that each patient will be given a treatment plan that would have been determined as nearly 100% effective because of their genetic makeup, anatomy, and physiology. Because of that, this approach fits perfectly into the concept of precision medicine—if an approach is truly rational, it can tell us whether a patient is going to respond to treatment before that patient has even been injected with the drug.

Key components of rational therapeutics are modularity and image guidance. Modularity allows us to synthesize libraries of therapeutic agents that are optimized for a given indication or a given patient in terms of size, surface coating and charge, hydrophilicity/hydrophobicity; antigen-targeting through incorporation of targeting peptides, and therapeutic moiety. Image guidance answers questions about the individual biology of a given patient or indication. It is necessary because we know that each organ and each patient are highly variable in terms of vascularity, fat content, vascular permeability, inflammatory profile, etc. Because of these differences, we need to vary features such as physicochemical drug design, injection dose, schedule, and even route, in order to provide the best and most effective treatment possible for every individual patient. Imaging is the only modality that can provide answers to these questions.

In our experience, an ideal candidate for rational drug design has three components: a DNA/RNA therapeutic component, a nanoparticle carrier, and an imaging reporter. DNA/RNA-targeted methods could be an integral part of rational therapeutics. This essential component takes advantage of the “coded” nature of the genome and transcriptome. Because of that, DNA/RNA-targeted methods provide an ideal platform for completely rational design of diagnostic and therapeutic agents based on the phenomenon of complementarity. This approach can be used while relying on recent advances in genome sequencing like antisense oligonucleotides [locked nucleic acid (LNA) oligos, antagomirs, miRNA sponges], small interfering RNA (siRNA) duplexes, or ribozymes. These molecules can be synthesized to target portions of the code that are aberrant in disease and thus the unique genome of the patient would in turn direct us to an equally unique cocktail of therapeutic agents. This constitutes a prime example of achievable individualized medicine.

Nanotechnology is another component of the envisioned rational therapeutic. Specifically, nanoparticles are carriers that can incorporate all three components of the drugs that we envision. They can easily be functionalized with oligonucleotides (antisense, siRNA, etc.) without interfering with the functionality of the oligos. Their design can be fine-tuned using standard synthetic chemistry, e.g., liposomes, iron oxide nanoparticles, etc. Finally, they can be labeled with an imaging reporter, e.g., radionuclide. An example is presented by polymer-coated iron oxide nanoparticles. By varying the ratios of coating to iron, the size of the nanoparticles can be adjusted. In addition, fairly standardized chemistry can be used to incorporate different coats onto the nanoparticles, e.g., dextran, carboxydextran, starch, polyethylene glycol (PEG), etc. These changes in size and surface coating result in very diverse biological fates, related to the agents’ pharmacokinetics and pharmacodynamics.

Image guidance is the third component of the envisioned drug design. By labeling the drug with an imaging reporter, we can monitor the delivery of the therapeutic agent to the tissue of interest. Image-guided delivery can be instrumental to assess and control delivery to the target tissues. Imaging can be used to determine the optimal drug design, delivery schedule, route, and therapeutic dose on an individual basis and to suggest alternatives should therapy fail in a given patient. In our view, dual-modality approaches, such as PET-MR or PET-CT would be optimal. PET-MR specifically could obtain near perfect spatial registration of molecular/functional positron emission tomography (PET) and anatomic/functional magnetic resonance imaging (MRI). This combination allows highly detailed anatomical images (MR) to be co-registered with PET images that have greater sensitivity and the capability for precise quantitation of local drug concentration.

## Oligonucleotide Design

Of the three elements of rational therapeutics, oligonucleotide development is by far the most advanced. Partly, the reason for this investment in the development of oligonucleotide therapeutics stems from the vast potential to address therapeutic challenges, including undruggable targets. Synthetic oligonucleotides include, among others, antisense oligonucleotides, mRNA oligos, siRNA, microRNA inhibitors or mimics, and more recently, long non-coding RNA modulators. Unlike biologics or small molecules, the listed synthetic oligonucleotides have the advantage of generally binding their targets through direct Watson–Crick complementarity. This fundamentally straightforward property provides an opportunity for rational, computational design of therapeutics, based on the simple knowledge of the target and its sequence.

Nevertheless, there is a list of challenges related to the design of synthetic oligonucleotides for therapy. These challenges relate to the fact that the chemical architecture of the oligonucleotide needs to be fine-tuned according to the requirements for favorable pharmacokinetics. This includes ADME, long-term stability, safety, and immunogenicity, and capacity to engage the target with high affinity and specificity without off-target effects. The latter is especially important when the oligonucleotide functions in the context of a multi-enzyme complex, as is the case for siRNA.

On the level of oligonucleotide architecture, these issues are addressed by rationally designed chemical modifications. Backbone and sugar modifications are two general strategies that have found application. The most commonly encountered backbone modification is the phosphorothioate linkage. The phosphorothioate (PS) linkage imparts significant resistance to nuclease degradation. However, in addition to this role, the PS linkage also has a major impact on oligonucleotide trafficking and uptake. Specifically, phosphorothioates can promote oligonucleotide binding to serum albumin (1) and effectively alter the pharmacokinetics and circulation half-life of the oligos. Phosphorothioate linkages also enhance the cellular uptake of oligos without a carrier for transfection (2). Finally, phosphorothioate linkages can have a reduced binding affinity for the target (3), necessitating further optimization to offset this effect and increase potency.

Such a level of flexibility can be achieved through sugar modifications. These include, among others: incorporation of 2′*-O-*Me units to increase affinity and nuclease stability (4, 5); the incorporation of LNA analogs (6, 7) to dramatically increase binding affinity, improve nuclease stability, and reduce immunogenicity; and the incorporation of a 2′*-O-*MOE [2′*-O-*(2-methoxyethyl)] modification to increase affinity and nuclease stability (8). A thorough review on the subject can be found in Ref. (8, 9).

Iterative computational methods are available to arrive at a candidate that has the required properties for a given target. Still, an element of empiricism can be involved, for example, when designing siRNA oligos to an mRNA of interest due to variability in the properties of the specific binding site on the mRNA oligo. Oftentimes, this is addressed by utilizing a cocktail of siRNAs.

Nevertheless, compared to methodologies for the design and selection of small molecule drugs or biologics, the degree of empiricism is somewhat limited, and originates from the fact that even short oligos have unique structures that impact their biophysical properties. In this context, we would like to highlight a recent publication, which reported on the development of an automated methodology for the prediction of the pharmacological properties of short DNA/LNA oligonucleotides. Namely, the authors apply quantum mechanical calculations to predict structures and electrostatic surface potentials for the oligos, which are primary determinants of interaction between molecules (10).

**Figure 1:**
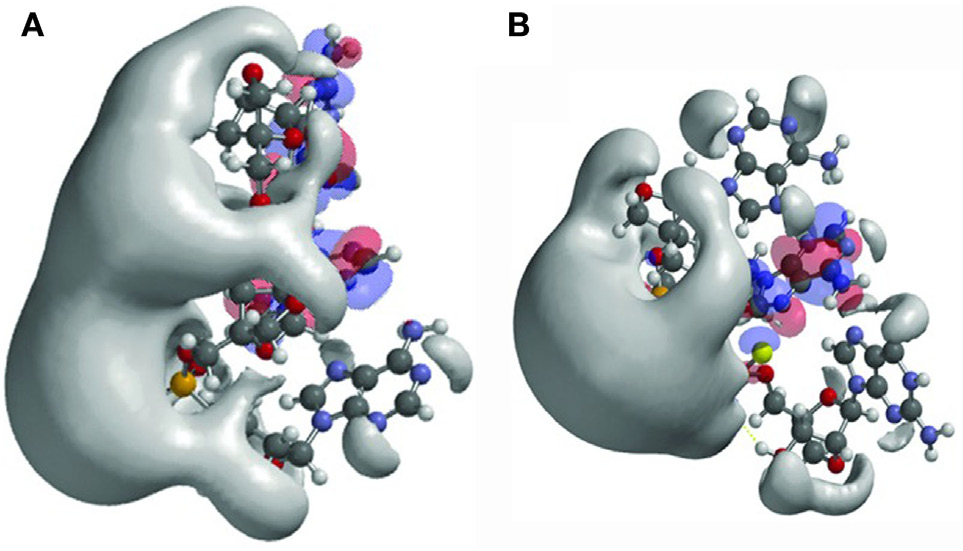
Structures and frontier orbitals (red/blue) for **(A)** locked nucleic acid (LNA) with the sequence 5′-AAG-3′ (5′ end at *bottom* of structure) and **(B)** LNA with sequence 5′-AAG-3′, chemically modified by phosphorothioate internucleoside linkages (PS). The electrostatic potential (negative) is shown in gray and represented by an *Iso*-potential value of −83 kJ/mol. Reproduced from Ref. (10) with kind permission by Mary Ann Liebert, Inc.

As an example, the authors studied the effects of changed internucleoside linkages from a phosphodiester (PO) to a phosphorothioate (PS). LNA-PO-AAG (Figure 1A) was compared with LNA-PS-AAG (with the PSs in the RS configuration) (Figure 1B). The LNA-PS-AAG (RS) modification resulted in a large potential change and exhibited a more scattered topology compared to LNA-PO-AAG, with a shift of the electrostatic potential toward the 5′ end. PS modification also induced changes in the localization of the frontier orbitals. The highest occupied molecular orbitals/lowest unoccupied molecular orbitals are split between adenine and guanine for LNA-PO-AAG (Figure 1A) but the LUMO resides on the central adenines for PS (Figure 1B) (10). The study illustrates the application of quantum mechanical modeling that can be used to understand antisense oligonucleotide properties and to explain the observation that small structural changes in oligonucleotide composition may lead to dramatic shifts in phenotypes, i.e., toxicity, protein binding, and tissue and cell uptake. This type of analysis could be applied in future oligonucleotide drug discovery and would allow the production improved antisense drugs.

## Nanocarrier Design

Despite the advancements in oligonucleotide design for therapy, rapid entry into the clinic is hampered partly by obstacles related to oligo delivery *in vivo*. After intravascular administration, oligos get aggregated by serum proteins in the plasma, and/or are rapidly eliminated by the kidneys, resulting in very short intravascular circulating half-lives in the range of minutes (11). Additional obstacles to delivery include passage across the vascular endothelium, diffusion through the extracellular matrix, and translocation across the cell membrane. Finally, oligonucleotides are associated with immune activation, further limiting their clinical application (12, 13).

These issues could be addressed by conjugating, complexing, or encapsulating the oligos to nanocarriers. These can be classified into three types: lipid-based, polymer-based, and inorganic nanoparticles. Nanocarrier design is optimized in terms of physicochemical properties (surface coating and particle size) in order to achieve optimal biodistribution and pharmacokinetics, to shield the oligo from degradation, to facilitate cellular uptake, and release the oligo in the proper intracellular compartment, and to satisfy requirements related to their physiological safety, including lack of immunogenicity, non-coagulation with serum proteins, and low non-specific uptake by normal tissues or cells.

As stated in Ref. (14), a major prerequisite for successful oligo delivery is the presence of positive charges on the surface of the nanoparticles that would facilitate uptake across cell membranes. However, cationic surface charges increase the chances of aggregate formation with plasma proteins. This can be overcome by the introduction of PEG on the surface of the nanocarrier. This can neutralize the surface charge and avoid interaction with plasma proteins, resulting in longer circulation half-life and faster diffusion across the extracellular matrix (15). However, this modification interferes with cellular uptake and necessitates an additional moiety for receptor-mediated endocytosis, which significantly limits the modular potential of the construct.

With respect to nanoparticle size, we are limited by the requirement that the size of the nanocarrier–oligo construct should be larger than the pore size of the glomerular filtering system (>7 nm) to avoid renal clearance but small enough (<100 nm) to avoid rapid phagocytosis by cells of the reticuloendothelial system (16–19). Typically, nanocarriers in the range of 10–100 nm in diameter are optimal to allow delivery to tissues through the enhanced permeation and retention effect. This is particularly relevant for solid tumors that are characterized by leaky vasculature (16, 20, 21). Even in poorly permeable tumors, nanocarriers smaller than 50 nm can penetrate the capillary endothelium, whereas micellar nanoparticles of 70 nm are retained in the vasculature (22). Based on these observations, the optimal size of nanocarriers for cancer treatment can be narrowed to 10–50 nm.

**Figure 2:**
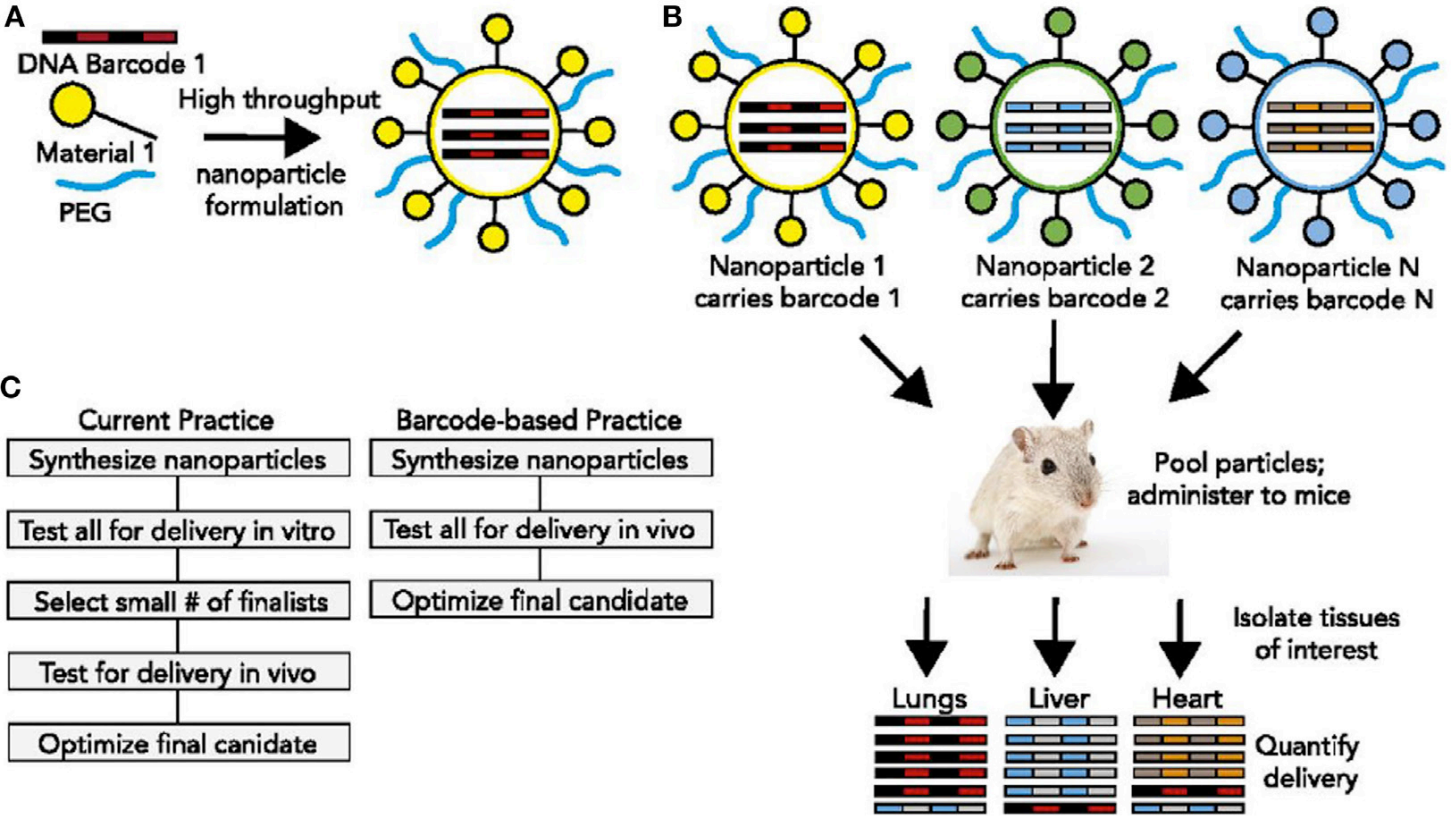
DNA barcoded nanoparticles for high-throughput *in vivo* nanoparticle delivery. **(A)** Using high-throughput fluidic mixing, nanoparticles are formulated to carry a DNA barcode. **(B)** Many nanoparticles can be formulated in a single day; each nanoparticle chemical structure carries a distinct barcode. Particles are then combined and administered simultaneously to mice. Tissues are then isolated, and delivery is quantified by sequencing the barcodes. In this example, nanoparticle 1 delivers to the lungs, nanoparticle 2 delivers to the liver, and nanoparticle *N* delivers to the heart. **(C)** This DNA barcode system enables multiplexed nanoparticle-targeting studies *in vivo*, improving upon the current practice, which relies on *in vitro* nanoparticle screening to identify lead candidates. Reproduced from Ref. (23) with kind permission by the National Academy of Sciences.

These requirements, as well as detailed descriptions of polymeric nanoparticles, lipid-based nanoparticles, and inorganic nanoparticles for oligo delivery can be found in Ref. (14). Here, we will highlight a few investigations that relate to the advancement of modularity in nanocarrier design. An ingenious new method was described earlier this year (23) that would permit the high throughput *in vivo* discovery of targeted nucleic acid nanotherapeutics. This methodology directly addresses the fact that nucleic acid therapeutics are limited by inefficient delivery to target tissues and by an incomplete understanding of how nanoparticle structure affects biodistribution. The authors developed a method to simultaneously measure the biodistribution of many chemically distinct nanoparticles by formulating the nanoparticles to carry specific DNA barcode oligonucleotides. These barcoded nanoparticles were then administered to mice *in vivo*. After a pre-selected time, nanoparticle biodistribution was quantified by deep sequencing the barcodes (Figure 2). This method permitted the accurate measurement of relative quantities of nucleic acid delivered to tissues and identified chemical properties promoting nanoparticle delivery to specific tissues (23). Using this system, it should be possible to rapidly select nanoparticles targeting specific organs following *in vivo* administration. The approach would also facilitate a deeper understanding of how the chemical structure of nanoparticles affects their delivery *in vivo*.

**Figure 3:**
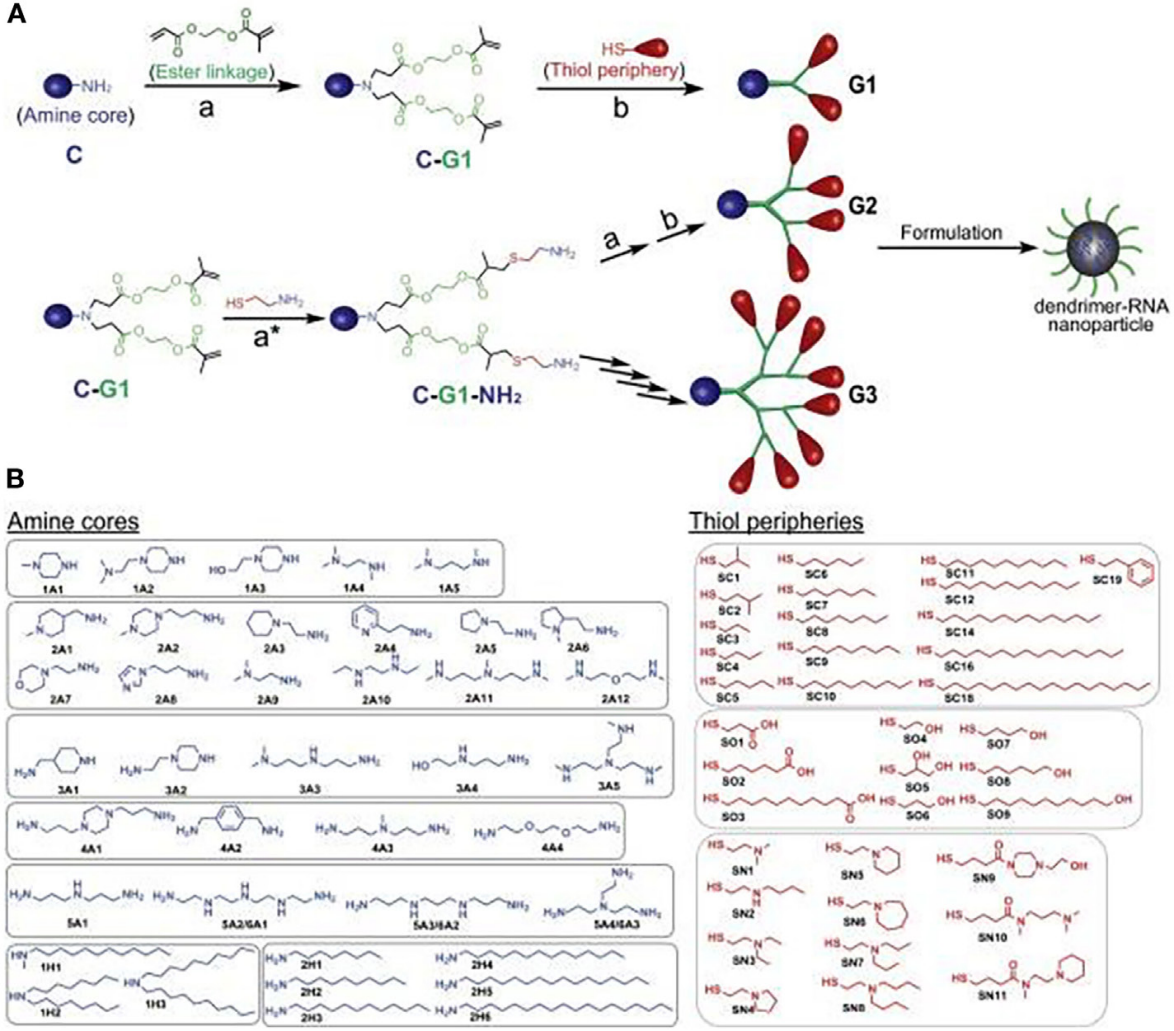
A modular strategy for diversifying the chemical functionality and size of ester-based dendrimers allowed discovery of potent and nontoxic dendrimers for *in vivo* small-RNA delivery to tumor cells. **(A)** Orthogonal reactions accelerated the synthesis of >1,500 modular degradable dendrimers by combination of 42 cores (C) and 36 peripheries (P) through degradable linkages (L) and generations. The library was established *via* sequential reactions. First, amines (C) with a series of N–H bonds reacted quantitatively and selectively with the less steric acrylate groups of AEMA (L). The products (C–L) then quantitatively reacted with various thiols (P) under optimized DMPP-catalyzed conditions. **(B)** Dendrimers were independently modulated with chemically diverse amines and thiols. Selected amines were divided into two categories: ionizable amines (1A–6A) to tune RNA binding from C that generated one to six branched dendrimers, and alkyl amines (1H–2H) to tune NP C stabilization. Alkyl thiols (SC1–SC19) and alcohol/carboxylic acid terminated thiols (SO1–SO9) were selected to tune NP P stabilization. Aminothiols (SN1–SN11) were selected to tune P RNA binding. G2–G4 higher generation dendrimers with multiple branches were also synthesized using generation expansion reactions. Reproduced from Ref. (24) with kind permission by the National Academy of Sciences.

To illustrate the modular potential of nanotechnology, we focus on a recent publication by Robert Siegwart’s group at the University of Texas Southwestern Medical Center (24). In their study, the authors synthesized more than 1,500 siRNA-incorporating modular dendrimers using sequential, orthogonal reactions where ester degradability was systematically integrated with chemically diversified cores, peripheries, and generations (Figure 3). They then screened these dendrimers in cell lines and in mice to identify candidates that achieve high potency to tumors and low hepatotoxicity and provide a pronounced survival benefit in an aggressive liver cancer model (24). This study is just an example of the power of nanotechnology for modularity. However, multiple nanocarriers, beyond dendrimers, could be synthesized using a modular approach. These include soft/hollow-core nanoparticles, such as lipid nanoparticles, polymeric nanoparticles, carbon nanotubes, and porous silica nanoparticles, as well as hard-core nanoparticles, such as quantum dots, gold nanoparticles, and iron oxide nanoparticles. Also, modular building blocks can be utilized for the optimization of nanocarrier structure in terms of size, surface charge, spacer length, and functional groups to ensure long-term stability, the enhanced interaction with target transcriptomes, and optimal circulation half-life/biodistribution.

## Imaging

The role of imaging in cancer therapy is well established. As part of their routine work-up, patients are imaged by ultrasonography, computerized tomography (CT), MRI, or positron emission tomography (PET) to both diagnose and stage the disease, and to monitor the progress of treatment. The value and necessity for this type of analysis is indisputable. In addition to imaging, a wide variety of circulating biomarkers, such as circulating tumor cells, exosomes, and circulating cell-free DNA/RNA, are finding application in recent years. Finally, more invasive *in situ* diagnostic methods that involve biopsy are widely used and largely considered as the gold standard diagnostic approach.

In this review, we focus on imaging not for the diagnosis, staging, and monitoring of cancer, but rather for the specific measurement of drug delivery to the target tissue and/or the overall pharmacokinetics of the drug. Even though this kind of studies are routinely involved in the process of drug development, they are not part of patient care. Since the overall goal is the modular development of strictly personalized therapeutic agents, in the treatment paradigm that we illustrate here, knowledge about pharmacokinetics and drug delivery need to be obtained for each patient. Hopefully, this could be accomplished concurrently with the assessment of therapeutic response.

**Figure 4:**
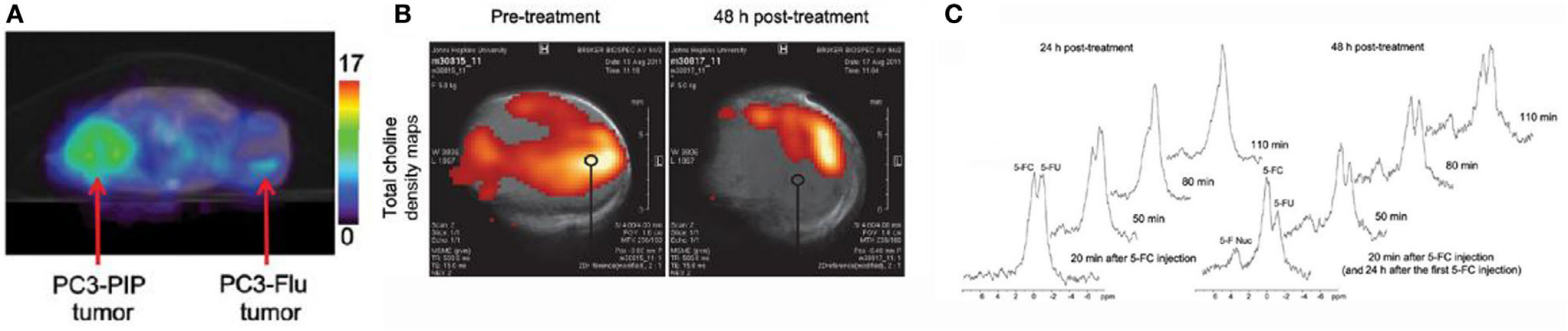
A multimodal approach for the non-invasive assessment of drug bioavailability and therapeutic effect in a model of prostate cancer. **(A)** Representative SPECT images of a SCID mouse bearing PC3-PIP and PC3-Flu tumors. **(B)** *In vivo* total choline density maps from 2D CSI datasets acquired from a representative PC3-PIPtumor (~400 mm^3^) before and 48 h after i.v. injection of the PSMA-targeted nanoplex 1 (150 mg/kg). **(C)** *In vivo* ^19^FMR spectra acquired from a PC3-PIP tumor (~400 mm^3^) at 24 and 48 h after i.v. injection of the PSMA-targeted nanoplex (150 mg/kg) carrying bCD and siRNA-Chk. Reproduced from Ref. (25) with kind permission by the American Chemical Society.

One study that illustrates this vision comes from Zaver Bhujwalla’s group at Johns Hopkins University (25). The authors developed a PSMA-targeted nanoplex platform for theranostic imaging of prostate cancer. The therapeutic nanoplex was designed to deliver siRNA against choline kinase (Chk) along with a prodrug (5-fluorocytosine; 5-FC). Importantly, the nanoplex contained multimodal imaging reporters that permitted the assessment of its accumulation in tumors and its therapeutic effect manifested as siRNA-mediated down-regulation of Chk and the conversion of the prodrug 5-FC to the cytotoxic drug 5-FU. Specifically, SPECT/CT of mice bearing PC3-PIP and PC3-Flu tumors revealed a significantly higher uptake of the targeted nanoplex in PSMA-overexpressing PC3-PIP tumors compared to PC3-Flu tumors (Figure 4A). To assess the efficacy of siRNA-Chk to downregulate Chk, the authors acquired *in vivo* 1H MRSI of PC3-PIP tumors 48 h after administration of the nanoplex. As shown in Figure 4B, there was a significant decrease of the total choline signal that consists of free choline, PC, and glycerophosphocholine. Moreover, by performing 19F MRS, the authors observed the activity of the prodrug enzyme bCD, as it converted the prodrug 5-FC to 5-FU (Figure 4C) (25).

In our own work, we have strived to incorporate imaging of drug delivery as an indispensable companion to drug development. We have extensively used dextran coated iron oxide nanoparticles as delivery vehicles for siRNA or antagomir therapeutics to tumors (26–31).

Since these nanoparticles are easily detected by MRI, we have utilized this modality to measure drug bioavailability *in vivo*.

**Figure 5:**
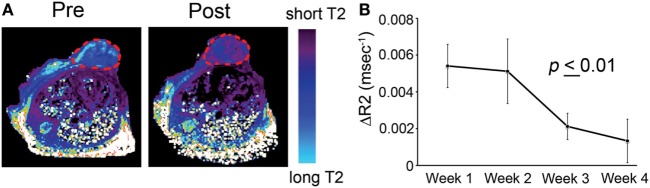
T2-weighted magnetic resonance imaging of MN-anti-miR10b accumulation in orthotopic MDA-MB-231-luc-D3H2LN tumors. **(A)** Representative color-coded T2 maps before (left) and 24 h after (right) MN-anti-miR10b injection demonstrating a shortening of the T2 relaxation times of the tumors (outlined) consistent with nanodrug accumulation. **(B)** Quantitative analysis of ΔR2 relaxation rates (1/T2 pre − 1/T2 post, ms) of the tumors, suggesting a tendency toward build-up of the MN-anti-miR10b (*p* ≤ 0.01, *n* = 12). Data are represented as mean ± SD. Reproduced from Ref. (29) with kind permission by Nature Publishing Group.

A specific application is illustrated in Ref. (29). The study described the testing of an imaging-capable nanodrug that was designed to inhibit the pro-metastatic miRNA-10b. The nanodrug’s physicochemistry was specifically optimized to reach primary and metastatic tumor cells. Therapy was delivered to a mouse model of metastatic breast cancer. During the course of treatment, the animals were imaged by MRI in an attempt to fine-tune the administration schedule and design of the nanodrug. MRI revealed that the nanodrug was successfully delivered to the tumors, as shown by the decrease in transverse relaxation time (Figure 5A). Quantitative analysis suggested a tendency toward nanodrug build-up in the tissue after the second treatment session (Figure 5B). These results illustrate a potentially useful application of imaging, especially when the delivery vehicle is innately imaging capable, as is the case for iron oxide nanoparticles. Such capabilities could be essential when optimizing a modular nanocarrier of this kind (29).

**Figure 6:**
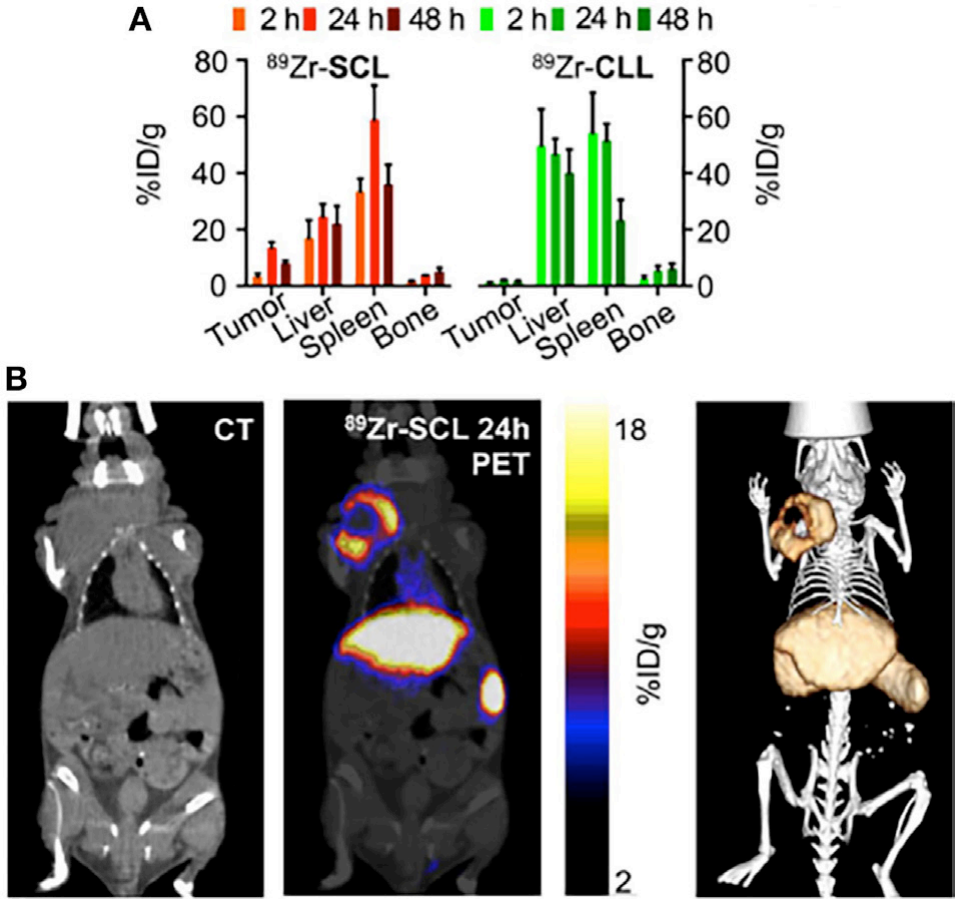
Modular labeling for *in vivo* positron emission tomography (PET) imaging of tumor-homing nanoparticles. **(A)** Radioactivity distribution in selected tissues of ^89^Zr-SCL and ^89^Zr-CLL. **(B)** PET/computerized tomography (CT) imaging of ^89^Zr-SCL: CT only (left), PET/CT fusion (middle), and 3-dimensional rendering of PET/CT fusion (right) at 24 h after injection. Reproduced from Ref. (32) with kind permission by the Society of Nuclear Medicine and Molecular Imaging.

When the nanocarrier is not innately imaging-capable, it is highly beneficial to apply protocols for modular labeling of the nanocarrier with a radionuclide for nuclear imaging. This approach is illustrated in a study by Thomas Reiner’s group at Memorial Sloan-Kettering Cancer Center (32). The authors developed an ^89^Zr-based labeling strategy for liposomal nanoparticles that accumulate in tumors *via* passive targeting mechanisms. Labeling was accomplished either by click labeling (CLL) or surface chelation (SCL), illustrating the versatility of the labeling process. PET/CT imaging of the radiolabeled nanoparticles was performed on a mouse model of breast cancer. Figure 6A shows a comparative ^89^Zr activity biodistribution in selected tissues after intravenous administration of the liposomes in mice bearing breast cancer xenografts. PET/CT imaging with ^89^Zr-CLL at 2 h after injection showed predominantly liver and spleen uptake. In contrast, ^89^Zr-SCL PET images at first showed high blood-pool activity and strong signals from liver and spleen. At 24 h, the blood-pool signal was moderate but tumor accumulation was higher (Figure 6B) (32).

The highlighted papers represent just a first step in the direction of integrating imaging more closely with the process of drug development and treatment planning. Given the need to obtain concurrent information of therapeutic response and drug delivery, it is likely that such approaches would involve a dual-modality capability, most likely PET-MR or PET-CT. The combination of these modalities is uniquely poised to obtain quantitative co-registered information about tumor dynamics, as a function of drug accumulation and could provide clues about the failure of some therapies in a given patient from the standpoint of drug delivery. On a broader scale, the introduction of this methodology would greatly enrich the process of drug development, which is currently hampered by a limited understanding of patient-to-patient variability in terms of drug delivery.

## Future Outlook

While exciting, the vision we outline may be criticized for being unrealistic. A major impediment to the fulfillment of this vision would likely be cost. It does not seem cost-effective to completely elucidate the genetic profile of a given patient’s tumor, identify causative therapeutic targets, and design oligonucleotide probes that would normalize the tumor cell phenotype. It would also not be cost-effective to diagnose and monitor each patient using sophisticated imaging modalities at multiple time points during therapy. Finally, the cost of synthesizing individualized drugs may seem overwhelming. However, given the high degree of trial-and-error empiricism that defines the current approaches, it is likely that the cost of multiple ineffective treatments is equally great not only in terms of money but, more importantly, in terms of patient distress and treatment failure.

An additional important point is the clear need for technological advancement before the vision of fully rational therapeutics could be fulfilled. Areas that need development include the following:

1. Complete elucidation of the genetic/epigenetic regulatory pathways that lead to cancer, so that we functionally understand the transcriptome of the cancer cell. This will allow us to predict the complete array of phenotypic effects of targeting a given gene/mRNA/or miRNA;
2. Design and validation of oligonucleotide probes that have the highest specificity and no off-target effects using non-empirical computational approaches;
3. Design and optimization of delivery vehicles for these oligonucleotide probes based on target organ, desired circulation half-life, need for no systemic toxicity, etc;
4. Development of standardized labeling protocols for image guidance and the implementation of optimal imaging protocols and instrumentation for a given modality.

Despite these issues, the vision of fully rational cancer therapy inspires hope defined mostly by the promise of better outcomes. Cancer is still largely an unaddressed health issue. It remains the second most common cause of death in the US, accounting for nearly one of every four deaths. As an example, pancreatic cancer is a devastating diagnosis defined by a mere 2% 5-year survival when diagnosed at an advanced inoperable stage, which defines 80% of the cases. Despite overall progress in research, the prognosis for people with pancreatic cancer has not improved in over 40 years. In light of the tremendous suffering caused by this disease and the modest progress achieved using standard treatments, it is clear that we need to explore radical, transformative approaches for therapy. The vision of fully rational drug design represents a hopeful step in that direction.

## Author Contributions

ZM conceived of the ideas presented and drafted the review. BY and A-MB drafted the sections detailing the highlighted research papers and provided feedback on the discussion.

## Conflict of Interest Statement

ZM is Founder, Director, and Scientific Advisory Board Member of TransCode Therapeutics, Inc.
